# Supportive Homes as Mediator between Rural Status and Disability of Older Adults

**DOI:** 10.1093/geroni/igab046.2395

**Published:** 2021-12-17

**Authors:** Bernard Steinman, Bremen Whitlock, Casandra Mittlieder, Julie Overton, Jon Pynoos

**Affiliations:** 1 University of Wyoming, University of Wyoming, Laramie, Wyoming, United States; 2 University of Wyoming, Laramie, Wyoming, United States; 3 University of Southern California, Los Angeles, California, United States; 4 University of Southern California, University of Southern California, California, United States

## Abstract

By definition, older adults living in rural communities have fewer formal resources available to address aging-related functional needs. Supportive environments are frequently relied on in rural settings to help address this discrepancy. The purpose of this study was to assess the role of supportive housing features and home modifications in mediating the association between rurality and disability. We hypothesized that environmental supports would be more crucial in rural settings than non-rural settings. We analyzed data from the National Health and Aging Trends Study (NHATS). Variable selection was guided by the International Classification of Functioning, Disability and Health (ICF), including covariates for sociodemographics, chronic conditions, mobility functioning, and participation. A series of regression models tested mediation by environmental variables of the association between rurality (as determined by the metro/nonmetro file indicator) and ADL/IADL disability. Supportive home environments were operationalized using indicators of whether participants had access to homes from the outside without having to use stairs; presence of a bedroom, kitchen, and full bathroom with a shower or tub on the same floor; and whether bathroom fixtures had been modified with features such as grab bars. Results suggest a statistical relationship between rurality and disability that is explained in part by the presence or lack of supportive home features, and these effects were greater in rural settings. Implications are that older adults who live in rural settings can benefit greatly by supportive environments and modifications in areas of the home that are known to cause difficulty.

